# Sperm Methylome Profiling Can Discern Fertility Levels in the Porcine Biomedical Model

**DOI:** 10.3390/ijms22052679

**Published:** 2021-03-06

**Authors:** Fabio Pértille, Manuel Alvarez-Rodriguez, Arthur Nery da Silva, Isabel Barranco, Jordi Roca, Carlos Guerrero-Bosagna, Heriberto Rodriguez-Martinez

**Affiliations:** 1 Department of Physics, Chemistry and Biology, Linköping University, SE-58183 Linköping, Sweden; fabio.pertille@liu.se (F.P.); arthur_nery@usp.br (A.N.d.S.); carlos.guerrero.bosagna@ebc.uu.se (C.G.-B.); 2 Department of Biomedical & Clinical Sciences (BKV), Linköping University, SE-58185 Linköping, Sweden; manuel.alvarez-rodriguez@liu.se (M.A.-R.); isabel.barranco@udg.edu (I.B.); 3 School of Agronomy and Veterinary Medicine, University of Passo Fundo, Passo Fundo RS 99052-900, Brazil; 4 Department of Medicine and Animal Surgery, University of Murcia, 30100 Murcia, Spain; roca@um.es; 5 Environmental Toxicology Program, Department of Organismal Biology, Uppsala University, 752 36 Uppsala, Sweden

**Keywords:** spermatozoa, genotyping by sequencing (GBS), methylated DNA immunoprecipitation (MeDIP), artificial insemination, fertility, prolificacy, pig

## Abstract

A combined Genotyping By Sequencing (GBS) and methylated DNA immunoprecipitation (MeDIP) protocol was used to identify—in parallel—genetic variation (Genomic-Wide Association Studies (GWAS) and epigenetic differences of Differentially Methylated Regions (DMR) in the genome of spermatozoa from the porcine animal model. Breeding boars with good semen quality (*n* = 11) and specific and well-documented differences in fertility (farrowing rate, FR) and prolificacy (litter size, LS) (*n* = 7) in artificial insemination programs, using combined FR and LS, were categorized as High Fertile (HF, *n* = 4) or Low Fertile (LF, *n* = 3), and boars with Unknown Fertility (UF, *n* = 4) were tested for eventual epigenetical similarity with those fertility-proven. We identified 165,944 Single Nucleotide Polymorphisms (SNPs) that explained 14–15% of variance among selection lines. Between HF and LF individuals (*n* = 7, 4 HF and 3 LF), we identified 169 SNPs with *p* ≤ 0.00015, which explained 58% of the variance. For the epigenetic analyses, we considered fertility and period of ejaculate collection (late-summer and mid-autumn). Approximately three times more DMRs were observed in HF than in LF boars across these periods. Interestingly, UF boars were clearly clustered with one of the other HF or LF groups. The highest differences in DMRs between HF and LF experimental groups across the pig genome were located in the chr 3, 9, 13, and 16, with most DMRs being hypermethylated in LF boars. In both HF and LF boars, DMRs were mostly hypermethylated in late-summer compared to mid-autumn. Three overlaps were detected between SNPs (*p* ≤ 0.0005, *n* = 1318) and CpG sites within DMRs. In conclusion, fertility levels in breeding males including FR and LS can be discerned using methylome analyses. The findings in this biomedical animal model ought to be applied besides sire selection for andrological diagnosis of idiopathic sub/infertility.

## 1. Introduction

Infertility is a complex disease, caused in approximately 50% of the cases by the male in the couple [1]. To complicate matters, about 15% of healthy men, despite having semen with acceptable numbers of intact and functional spermatozoa in terms of normal sperm nuclear DNA fragmentation and seminal oxidative stress [1,2] are yet sub/infertile and diagnosed as idiopathic infertile [3]. This unexplained male subfertility is also seen in livestock, such as pigs, which is a species considered a useful animal model for biomedical research for its increasing recognition of anatomical and physiological similarities with human [4]. As an example, ejaculate composition and ejaculation modes bear ample similarities, as we have studied over the years [5,6,7,8,9]. Commercial pig production has benefitted from the widespread use of artificial insemination (AI) of semen collected from genetically selected breeding boars. The boars undergo rigorous reproductive controls to ensure that they produce ejaculates with a large number of viable and motile spermatozoa [10]. Despite these selection criteria, between 5 and 10% of the highly selected breeding boars show fertility outcomes below the breed average and are thus considered subfertile [11]. This unexplained subfertility had led to calls over the years for more sophisticated semen analyses, aiding elimination of clearly subfertile sires [12]. Considering that the spermatozoa carries intact, healthy attributes that could define fertility when retrospectively related to the observed sire fertility [12], novel methods include omics of spermatozoa [13,14,15] and seminal plasma (SP, [7]), which is a trend also used in human andrology [16]. However, reliable prognosis for fertility in either species has remained elusive, often because the definition of fertility levels for a given male must indeed consider a plethora of confounding factors, including number of inseminated spermatozoa, intercourse frequency and, in pigs, an even number of females undergoing AI, number of AIs per oestrus, seasonality, the systems used to detect oestrus, farm characteristics, AI procedures, etc. All these factors must be systematically and statistically tested, alongside comparisons within breeding lines, assuming that genomics affects fertility [17].

The contribution of genetic effects to specific traits has for quite some time been explored using Genomic-Wide Association Studies (GWAS) aiding the identification of candidate genes associated with semen traits [18], including sperm number, sperm motility, and morphological abnormalities [19,20]. These studies are highly relevant, despite the multifactorial character of fertility, and they assume that the intactness of the chromatin is preserved [21]. Fertility is not only related to chromatin integrity but also to modifications in epigenetic factors that could explain some of the idiopathic male subfertility cases [22]. These include post-translational events such as histone modifications, the action of non-coding RNAs [9,23] and DNA methylation of CpG dinucleotides [21,24], events that—concerted—can imply intergenerational inheritance via spermatozoa [25].

The present study postulated that epigenetic differences in the spermatozoa of pig—a most relevant animal for biomedicine [4]—not only predict an altered state in the spermatozoa but further correlate with fertility beyond the genomic differences that selection lines establish and the display of good semen quality. 

Since the methylome of ejaculated spermatozoa can provide cues for the prognosis of fertility, spermatozoa from AI breeding boars, pre-selected for semen quality (yet displaying specific and well-documented differences in fertility and prolificacy) alongside boars with unknown fertility were explored for epigenetic changes through a combined Genotyping By Sequencing (GBS) [26,27,28]) and methylated DNA immunoprecipitation (MeDIP) technique [27,28,29]. This methodological protocol allowed parallel identification of genetic and epigenetic differences between experimental groups in the same reduced fraction of the genome across individuals, i.e., identifying Single Nucleotide Polymorphisms (SNPs) and Differentially Methylated Regions (DMR). This method enabled the assessment of genome-wide levels of methylation in a reduced fraction of the genome that is not biased toward CpG islands, by using a restriction enzyme unrelated to CpG sites. The study also considered seasonal differences in the production of the AI sperm doses used.

## 2. Results

The statistical tests for the genetic analysis were made by comparing four High Fertile (HF) with three Low Fertile (LF) using GWAS through a weighted Mixed Linear Model (MLM). For the epigenetic analysis, we used a Linear Model to test for differential methylation between five defined contrasts, described in Table 1.

### 2.1. Identification of SNPs

**Figure 1:**
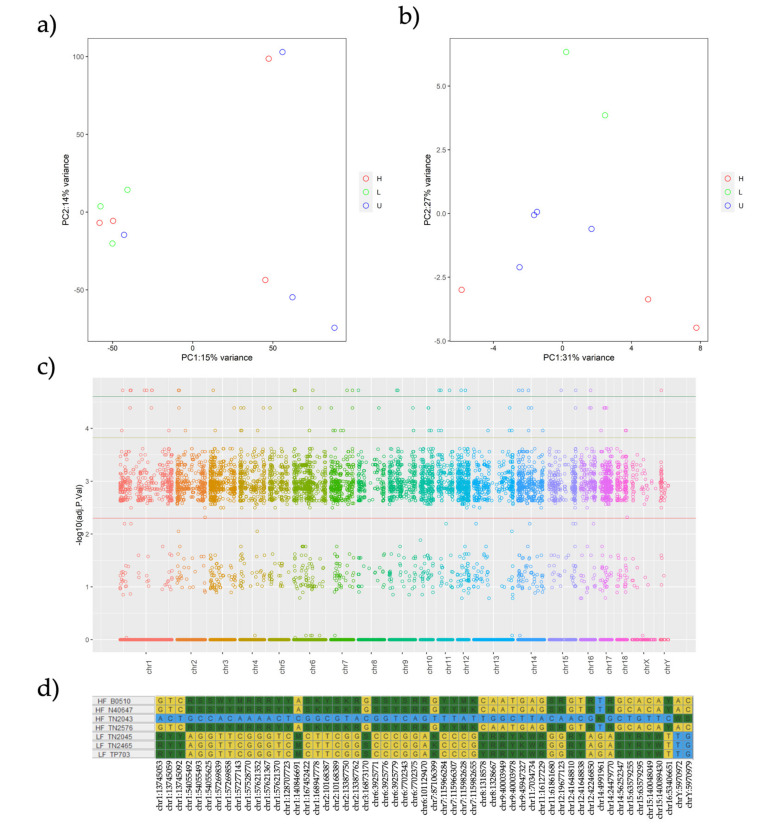
(**a**–**d**). Principal Component Analysis (PCA) across 11 individual boars from High Fertile (HF), Low Fertile (LF), and Unknown Fertility (UF) experimental groups depicting in (**a**) all 42,963 Single Nucleotide Polymorphisms (SNPs) and in (**b**) the significant 169 SNPs; (**c**) is a Manhattan plot of all the analyzed SNPs with the green threshold separating the TOP SNPs from the others. Finally, (**d**) lists the loci frequency of these TOP SNPs among all the analyzed boars.

A total of 165,944 SNPs were identified among all 11 individuals assayed, based on input DNA (Supplementary Figure S1a,b). All individuals remained after applying a sample call rate ≤ 90% and a loci call rate ≤ 70%, which resulted in 90,678 SNPs being kept for further analysis. Figure 1a shows the separation of the individual boars among selection lines based on Principal Component Analysis (PCA). Expectedly, PCA analysis generated two eigenvalue factors, PC1 explaining 15%, and PC2 explaining 14% of the variance (Figure 1a), thus confirming the investigated boars did not exhibit genetic sub-structures between individuals.

### 2.2. Genome-Wide Association Analysis (GWAS) for Boar Fertility

For the GWAS performed between HF and LF boars (*n* = 7, 4H and 3L), 87,219 SNPs with a total genotyping rate of 0.93 and an adjusted genomic inflation “est.lambda” (based on median chisq) of 1.78 were used. This implied 5630 SNPs were eliminated from the total of 90,678 SNPs, and they were considered heterozygous for all the individuals analyzed. Further, 47,717 SNPs that exhibited no genetic effect (additive or dominant) were eliminated from further analyses. On the remaining 42,963 SNPs, a Bonferroni multiple test correction (*p*-Value ≤ 0.0005) was applied, which yielded 1318 different SNPs between the HF and LF boars. Complete information on statistics and annotations performed for each one of these tested SNPs is available in Supplementary Spreadsheet S1. Since this threshold did not clearly separate the HF from LF boars in the PCA (Supplementary Figure S1a), we lowered the *p*-Value threshold to *p* ≤ 0.00015, thus obtaining 169 SNPs that produced an evident separation of the groups (Figure 1b). The distribution of SNPs across the chromosomes and the threshold *p*-Values used are shown in Figure 1c. 

For visualization purposes, Figure 1d shows the TOP SNPs with *p* ≤ 0.000025 (55 SNPs above green threshold line in Figure 1c) and their allelic changes between HF and LF boars. There were no evident differences in the PCA results originated using this last threshold, as indicated in the Supplementary Figure S1b. Independent of the threshold used, the Unknown Fertility (UF) boars did not fit as a group, either with confirmed HF or with LF boars after the PCA. Principal Components (PCs) from the significant SNPs explained 58% of the variance among all boars.

From the 55 TOP SNPs selected as potential candidate genes controlling functions that distinguished HF from LF boars, 30.9% were located on Chr1. Based on the pig reference annotation, 35 SNPs (63.64%) were located in intronic regions, followed by 12 SNPs located in distal regions (21.82%) and eight SNPs located on promoters (14.55%) (Table 2). In addition, five SNPs were classified as CpG-SNPs and are located in intronic regions. These are located in the known genes *SERPINA3-2*, *CFAP99*, *FARP2*, and in two novel genes (*ENSSSCG00000004081*, *ENSSSCG00000039894*). Four of these CpG-SNPs show a higher frequency of the C allele in LF than in HF boars. In the reference pig genome, four of these CpG-SNPs are CpGs and one is a GpG (Table 2).

### 2.3. Putative DMR Identification: Does Fertility Vary with the Season of Semen Collection?

A total of 7815 DMRs by region of interest (ROI) peak calling between HF and LF boars were identified, counting the number of reads for each of the 11 individuals and their replicates, totalizing 39 measurements (Supplementary Table S1).

**Figure 2:**
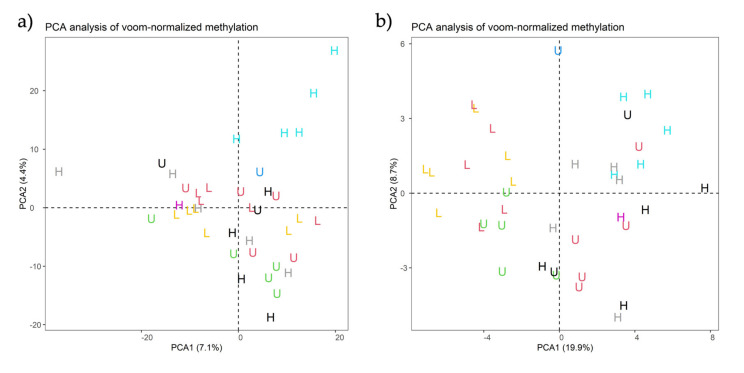
(**a**,**b**). Principal component analysis (PCA) of (**a**) all 1209 regions of interest (ROIs) and (**b**) the 46 significant Differentially Methylated Regions (DMRs) across 39 samples from HF (*n* = 15), LF (*n* = 11), and UF (*n* = 13) experimental groups represented by the letters H, L, and U, respectively.

**Figure 3:**
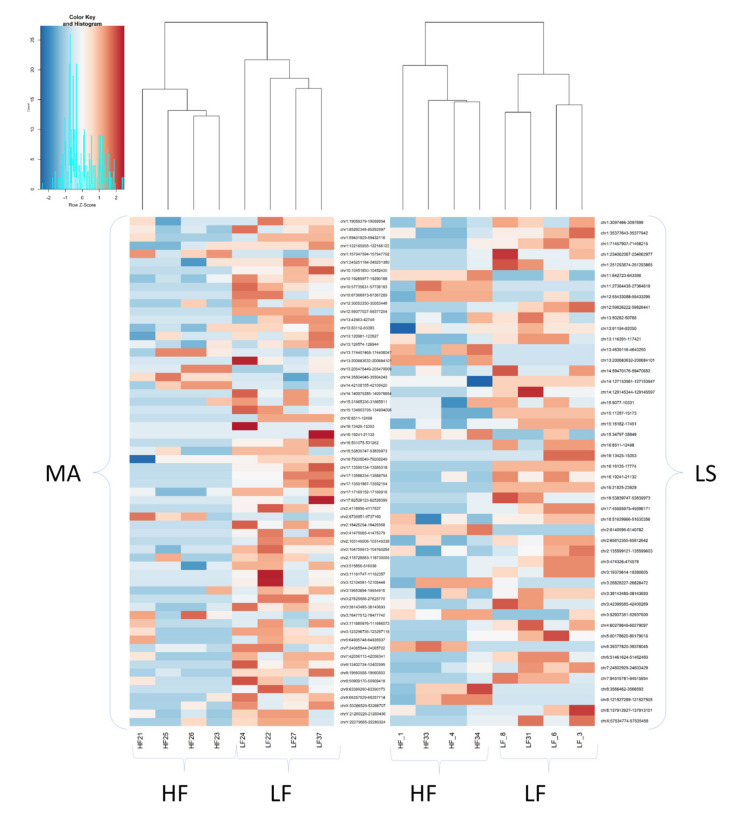
Heatmap across 39 samples from boars ranked as high fertile (HF, *n* = 15) or low fertile (LF, *n* = 11). LS = late-summer, MA = mid-autumn.

When using a minimum row sum (minRowSum) threshold of five for the reads counted across the 39 analyzed measurements (15 HF, 11 LF, 13 UF), a total of 1209 ROIs were identified and considered as putative DMR in the spermatozoa between the experimentally defined contrasts indicated in Table 1. The PCA analysis resulted in two eigenvalue factors wtih PC1 explaining 7.1% and PC2 explaining 4.4% of the variance (Figure 2a) when using “duplicateCorrelation” analyses and different effects as covariates in the model such as collection week, collection code, and collection period (Supplementary Spreadsheet S2). The analyses indicate that these covariates did not affect the model when fertility was used as an independent variable, prompting the screening of Fertility and Period of semen collection as fixed effects in an independent linear model for each test (Table 3). The intention was to sub-set boars considering Fertility and Period of semen collection at the same time. We identified more DMRs between HF individuals across different seasons than between LF boars (Table 3). Moreover, the DMRs were mainly hypermethylated during the LS season (Figure 3). The PCA of Figure 2b, where PC1 explained 19.9% and PC2 explained 8.7% of the variance (Figure 2b), shows that the replicates of the same individuals are scattered across the graph and not clustered in one region, which indicated that time collection affected the sperm methylation of the individual. Interestingly, in this scenario, the UF boars appeared clearly clustered with either one of the groups (HF or LF)(Supplementary Figure S3). Using the same color classification as the previous graph, we plotted PCAs with the significant DMRs for the other three variables (“collection code”, “collection period”, and “week”) to confirm they are independent variables when comparing with fertility (Supplementary Figure S2).

### 2.4. The Largest Differences between Highly Fertile (HF) and Low Fertile (LF) Boars Are Present in Few Chromosomes

**Figure 4:**
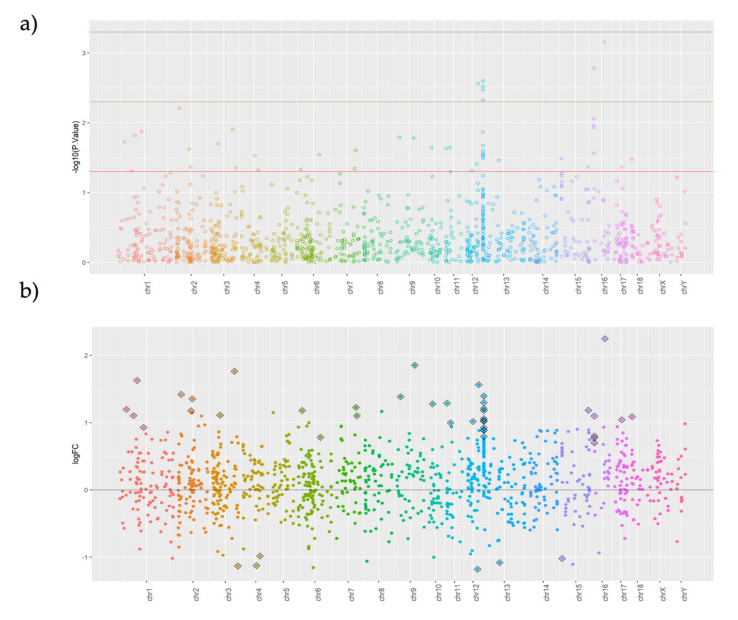

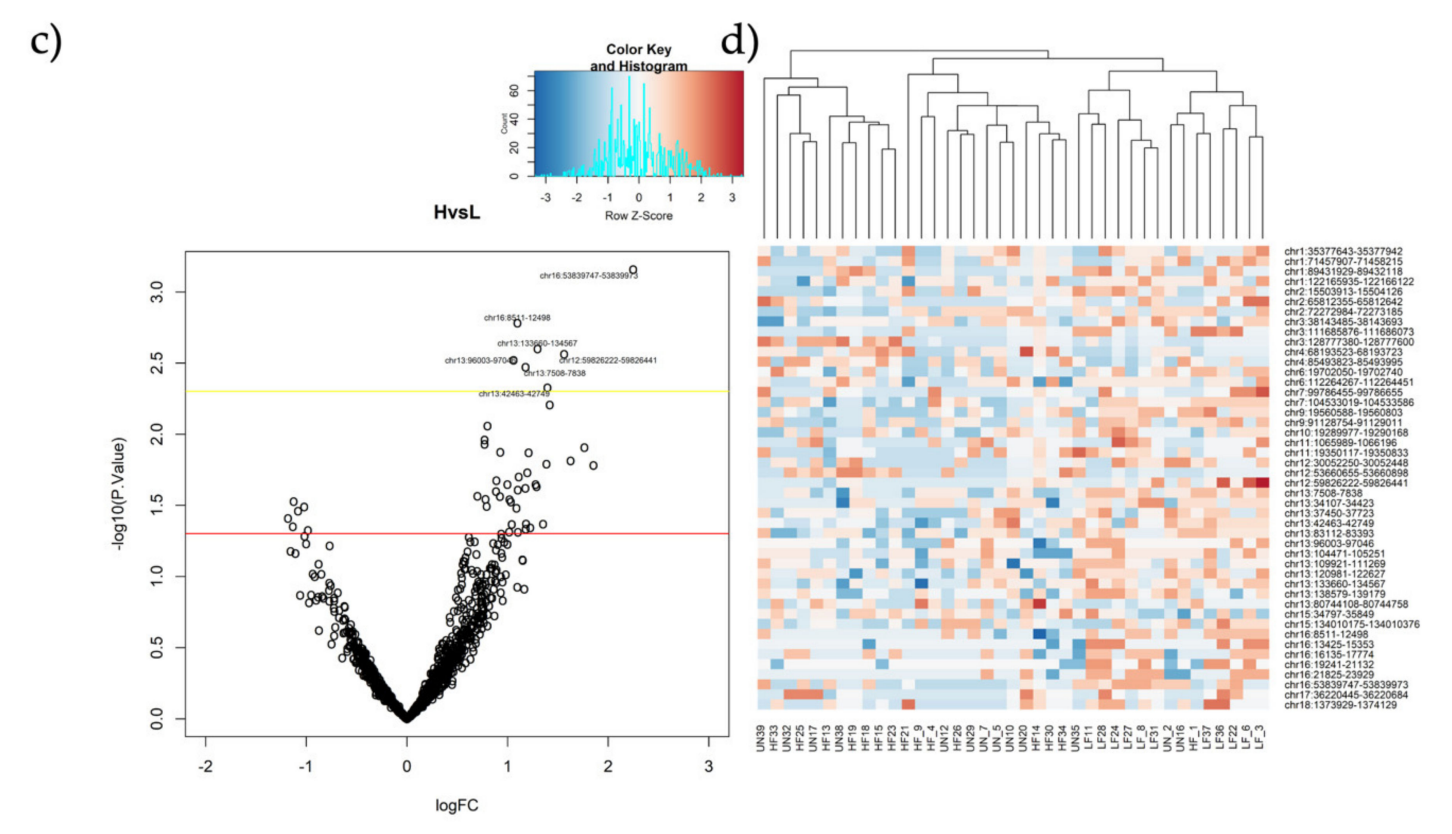
(**a**–**d**) Manhattan (**a**) and (**b**), volcano (**c**) plots, and heat map (**d**) showing fertility-related DMRs found between HF and LF experimental boar groups. Plots represent −log 10 of *p*-Values (**a**) and fold-changes (**b**); the diamond (**b**) and heatmap represents only significant DMRs (**d**), which is also represented by the red threshold (**a**).

The Manhattan plot in Figure 4a shows two well-defined peaks located in chr 13 and 16, providing a better visualization of the Differentially Methylated Regions (DMRs) observed across the pig genome between the HF and LF experimental groups. In addition, Figure 4b reveals that the largest differences between HF and LF were located in chr 3, 9, and 16.

### 2.5. Most DMRs Are Hypermethylated in Low Fertility Boars

As depicted in the volcano plot (Figure 4c), all but six DMRs (*p* < 0.05) in LF boars were hypermethylated (Table 4). The heatmap in Figure 4d shows that the HF boars were clearly separated from the LF boars by the hierarchy clusters formed by the 46 significant DMRs, with only one exception (sample HF_1). When comparing LS vs. MA, in both HF and LF boars, the DMRs were mostly hypermethylated. The number of DMRs in HF compared to LF boars was similar across seasons (48 DMRs in LS and 62 DMRs in MA).

We identified 48 significant DMRs between HF and LF groups (Table 4) related to the following genes: THEMIS, FAM227B, U6, ADCY9, IFT172, RNF144A, VXN, LMX1A, KIFC3, TMEM126B, RAPGEF5, XPO4, ITM2B, KIF2B, ULK2, CLSTN2, ROPN1L, FOXI1, NOL4L, and ssc-mir-153.

### 2.6. Genomic Annotation of the SNPs and DMRs

**Figure 5:**
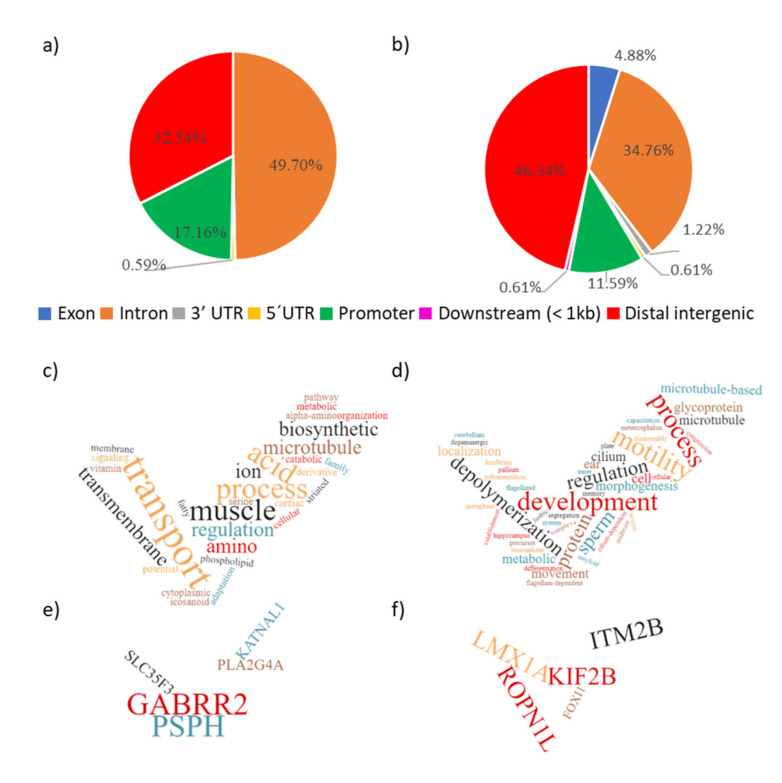
(**a**–**f**). Pie charts representing the functional annotation of the significant (**a**) SNPs and (**b**) DMRs, identified between HF and LF experimental boar groups. The work clouds represent the most frequent words that showed up though the significant Gene Ontology (GO)-pathways (adj *p* ≤ 0.2) of enriched genes related to the significant SNPs (**c**) and the DMRs (**d**); while the most frequent genes related with the identified pathways appear in (**e**) for SNPs and in (**f**) for DMRs.

The significant SNPs were mainly located intronic (49.70%) followed by distal intergenic (32.54%), in the promoters (17.16%) and 5’UTR (untranslated region, 0.59%) of the pig genes (Figure 5a). Of the 169 significant SNPs identified, 39 (23.07%) were located within CpG dinucleotides (as defined in the pig reference genome). From these 39 SNPs located at CpGs, 22 were located in the “cytosine” of the CpG (see Supplementary Spreadsheet S2 for detailed information). The location of the significant DMRs in the pig genome was quite similar to that of SNPs (Figure 5b). The DMRs were mainly located at distal intergenic (46.34%) regions, followed by introns (34.76%), promoters (11.59%), exons (4.88%), 3′UTR (1.22%), 5′UTR (0.61%), and downstream of genes (0.61%). The 49 significant DMRs presented 13.9 CpGs sites on average (see Supplementary Spreadsheet S3 for detailed information).

Genes related to significant SNPs and DMRs were tested with the enrichment GO analysis. The SNP-related genes appeared enriched in general cellular processes of regulation and transport of different components related to muscle hypertrophy (Figure 5c). The SNP-related genes that appeared the most in the pathways were *GABRR2*, *PSPH*, *SLC35F3*, *PLA2G4A*, and *KATNAL1* (Figure 5e and Supplementary Spreadsheet S4). In turn, DMR-related genes were associated with processes related with sperm development, motility, capacitation, and morphogenesis. Other pathways appeared, which are related with the development of the nervous system (Figure 5d), with DMR-related genes most identified as *ROPN1L*, *KIF2B*, *IRM2B*, *LMX1A*, and *FOX11* (Figure 5e, Supplementary Spreadsheet S4).

### 2.7. Overlaps between SNPs and DMRs

Using a *p*-Value threshold of *p* ≤ 0.0005 for the SNPs (Bonferoni; 1318 SNPs obtained) and of *p* ≤ 0.05 for the DMRs, we found three overlaps between SNPs and DMRs. These three overlaps were located in the same chromosomes previously pointed as DMR hotspots for fertility (chr13, 15, and 16). The first overlap encompassed a 1213 bp long DMR located at chr13:140,482-141,695, which contains 19 CpGs. This DMR is located 147,775 bps downstream of a novel gene (*ENSSSCG00000046931*). In this DMR, four SNPs were identified (chr13:141224 A > C, chr13:141230 C > G, chr13:141238 A > T, chr13:141241 T > A; HF > LF) between HF and LF boars. The second overlap corresponds to a 3886 bps long DMR located at chr15:11,287-15,173, containing 84 CpGs. This DMR is in an intergenic position located 24,846 bps upstream from a novel gene (*ENSSSCG00000047217*). In this DMR, we identified one SNP (chr15:14975 C > A; HF > LF) in which the allele C is the reference, and it is fixed in HF boars. However, its allele frequency in LF boars was only 37.5%. The third overlap identified corresponds to a 1928 bp long DMR located at chr16:13425–15353, containing 45 CpGs. This DMR is 28,000 bps upstream the *ROPN1L* gene. In this DMR, we identified one SNP (chr16:13620 T > C; HF > LF) exactly within a CpG position, according to the pig reference genome. For this SNP, LF boars had the “C” allele from the CpG in a higher frequency than HF boars (62.5% vs. 37.5%).

## 3. Discussion

The present study aimed to identify genetic variation (GWAS) in parallel with epigenetic differences in Differentially Methylated Regions (DMR) within the genome of spermatozoa from AI-breeding boars with good semen quality and specific and well-documented differences in fertility (farrowing rate) and prolificacy (litter size), by using a combination of GBS and MeDIP protocol. The study included a group of boars of Unknown Fertility (UF) as a test group to test whether the analysis could discriminate them according to previously found fertility-related epigenetic marks in the HF or LF boars. As well, it considered the period when the ejaculates were collected for the production of the AI doses used to measure fertility.

The GWAS has been previously used to aid the identification of genetic variants underlying reduced male reproductive performance as well as of candidate genes for semen traits in human [22,30], livestock [30,31], as well as in more suitable animal models, as pigs [14,15]. In either case, the rationale behind was to improve the identification of males suffering from idiophatic sub/infertility, despite providing ejaculates with values considered “within normal limits” [32,33]. Pigs, despite species differences with human, show similar hurdles [11]. The use of commercial pig AI provides fertility recordings of high accuracy, since ejaculates from individual boars produce tens of semen doses to inseminate sows, whose farrowings and litter sizes are accounted for [11]. Pigs, being suitable animal models for human, provide thus the basis for comparative studies, linking GWAS for the identification of specific semen traits, as morphology [16] or, ultimately fertility as hereby explored.

To the best of our knowledge, the present study is the first one reporting such differences in DMR in relation to sperm fertility. For that purpose, a combined GBS + MeDIPs methodology was used [27]. Two approaches were used for the analyses of the data: firstly, a DMR call across 100 bp windows (named ADJW), and secondly, pre-determined windows were used for the DMR call based on previous peak calling between the treatments (named ROI) [27]. In addition, the reduced genome of the boars was sequenced using the GBS approach [26] to analyze their genetic background. A simple PCA analysis of the almost 100,000 SNPs was not able, despite using restriction analyses that explained almost 60% of the variance, to allocate males with unknown fertility closer to the well-identified groups of respectively high-fertility and low-fertility boars. This is not unusual, the problem being previously seen in human studies [34] where the studied populations were usually burdened by many confounding factors, thus demanding further analyses.

Our approach was different, and for the GWAS, we only compared the HF and LF boar groups, since confounding factors that could affect fertility were already examined when selecting these boars. The UF boars could also be classified close to either one of the fertility-proven groups, suggesting a certain prognostic value for the methdology used. The GWAS analysis identified 35 SNPs (63.64%) in the intronic region based on pig annotation. Exon–intron boundaries have a described role in guiding the splicing machinery [35]. However, DMRs between exons, splice sites, and flanking–intronic regions are reportedly involved in the regulation of alternative splicing [36]. Moreover, three SNPs were classified as CpG-SNPs: *SERPINA3-2*, *FARP2*, and *CFAP99*, the latter related to flagella-associated protein. Regarding the CpG-SNPs, SERPINA3-2, a serine protease inhibitor involved in several functions including inflammatory response, has been studied at the genomic level [37]. In addition, the FMRFamide (Phe-Ile-Arg-Phe-NH(2))-related peptide, including FARP2, is known to affect opioid receptors, resulting in the elicitation of naloxone-sensitive antinociception and reduction of morphine-induced antinociception [38].

Four of these CpG-SNPs show a higher frequency of the C allele in LF than in HF boars. In addition, four of these CpG-SNPs are CpGs in the reference pig genome and one is a GpG, indicating that this CpG is liable to methylation. Moreover, if it occurs in an intronic region of the gene, it may be related to increased genetic transcription. It is known that methylation in regulatory regions (e.g., promoters) are usually associated with transcriptional repression, while in gene bodies, DNA methylation is associated with high levels of gene expression [39,40,41]. Therefore, the expression of the gene in the LF group where the CpG exists has the potential to increase if this CpG is methylated compared with the HF ones that have no CpG at that same position. In other words, it can have a DNA methylation differing between the groups that consequently could have a transcriptional effect between them. This is a potential genetic and epigenetic marker in the DNA to be further studied.

In the present study, we investigated DMRs emerging from different *p*-Value thresholds between HF and LF boars within each seasonal period of semen collection (i.e., LS: late-summer, LA: late-autumn, and MA: mid-autumn) in order to disclose if fertility varied with this variable. Results from the different statistical approaches showed an increase in differences, when it comes to seasons, in the HF boars, and also more differences between HF and LF boars during LS than in MA. Interestingly, 87 significant DMRs were found when comparing LS and MA in the high-fertile boars. This event is consistent with seasonal changes in semen quality under tropical environmental conditions, as in Brazil [42], but it has also been seen in the Mediterranean areas [43,44] where the boars under study have been raised, despite being reared under environment-controlled conditions.

The DNA methylation profile of gametes is increasingly used for its described relevance not only for embryo development but also during post-natal events [45]. Many studies in sperm DNA methylation have highlighted the relevance of this analytical tool to understand the plausible effect of these changes in the offspring [46] and also in relation to sperm quality [47]. However, boar sperm methylation analyses often forgot to include the female part when it comes to farrowing ratio and litter size. In this sense, we analyzed HF and LF boars, where all boars presented very high general quality semen parameters but higher or lower fertility success. 

A recent study has shown that DNA methylation patterns vary in boar spermatozoa in relation with DNA fragmentation [21]. Nevertheless, our study included only high-quality semen among all boars which, following the elimination of all possible confounding factors, yet depicted systematic and significant differences in fertility. Differences in DNA methylation patterns had been found in the IGF2-H19 locus, between Landrace and Large White boars [48]. As for the stability of the paternally derived cytosine methylation, DNA methylation reprogramming appears to be a non-conserved mechanism during early mammalian development [49]. Finally, the quality of the oocyte maturation determines the capability of reprogramming of the male chromatin into the male pronucleus [50].

In our study, most of the DMRs (42 out of 46) were hypermethylated in LF boars, and the highest differences between HF and LF boars were located in chr 3, 9, and 16. Whether these differences occur in other species or even in different cells or tissues should be tested in future studies. The 48 significant DMRs between HF and LF groups related to 20 different genes: *THEMIS*, *FAM227B*, *U6*, *ADCY9*, *IFT172*, *RNF144A*, *VXN*, *LMX1A*, *KIFC3*, *TMEM126B*, *RAPGEF5*, *XPO4*, *ITM2B*, *KIF2B*, *ULK2*, *CLSTN2*, *ROPN1L*, *FOXI1*, *NOL4L*, and *ssc-mir-153.* Some of these genes, such as *ROPN1L* [51] or *LMX1A* [52], are related to sperm motility, while *FOX11* was associated to sperm numbers and with processes related with sperm development, motility, capacitation, and morphogenesis [53], as discussed below.

Most of the genes related to fertility were hypermethylated in LF boar spermatozoa (leading to a plausible significant reduction in the genes included in the methylated region) and with a large variety of mechanistic functionalities directly or indirectly related to spermatogenesis, sperm function and quality, fertilization, and fertility/prolificacy. The *IFT172* (hypermethylated in our study) has been related to defects in spermiogenesis and infertility of male mice [54]. The hypermethylated *KIFC3* and *KIF2B* are also related to spermatogenesis [55,56] being testis-enriched, based on UniGene [57]. Moreover, the miR-26a supressed autophagy in swine Sertoli cells by targeting the hereby hypermethylated *ULK2* [58], together with *TMEM126B*, which is involved in anti-apoptotic functions in other animal models [59]. Thus, it is logical to consider that variations of these genes modifying sperm production and function could be linked to lower farrowing rate and litter size.

Sperm function can be modified by the expression of other genes, such as the *U6* gene. In Holstein bulls, some detected *U6* clusters were located in RNA, in relation to sperm traits (total motility and kinetic parameters, membrane integrity) [60]. In this sense, the *RAPGEF5* (hypermethylated in LF) is a gene that regulates nuclear traslocation of b-catenin [61] in the acrosome region of mouse epididymal spermatozoa [62] and might be involved in the acquisition of sperm motility—a sperm attribute that is clearly fertility-related. Relevant is the hypermethylated *ADCY9* gene which, being involved in cyclic AMP (cAMP) signaling, might rule sperm function [63]. *ROPN1L* relates to sperm motility via regulation of the two crucial proteins ROPN1/ROPN1L, which are associated with PKA/A kinase activity [64]. The loss of ROPN1L causes a defect in murine sperm motility [51]. Moreover, in human, the sperm protein 17 (Sp17) has also a regulatory role in the protein kinase A (PKA)-independent A-kinase anchoring protein (AKAP) complex, which is perhaps due to the high expression in the spermatogenic cells [65], and it might highlight the relevance of PKA in sperm motility-related events. The A-kinase anchoring protein-associated protein (ASP) has been described both in human spermatozoa and in the seminiferous tubules [66]. The *ITMB2B* gene function is associatied with *Adam7*, which is remarkably promoted through conformational changes during sperm capacitation [67]. It is noteworthy that the lack of *Adam7* in mice has resulted in a reduction on fertility [68]. A disregulation of sperm *miR-153* (here hypermethylated) ocurrs also in human, affecting fertilization and embryo rates after in vitro fertilization IVF [69], which is perhaps in relation to the function of *THEMIS* [70]. In addition, the forkhead box I 1 (*FOXI1*) appears crucial for male fertility, being involved in the reduction of the number of spermatozoa effectively reaching the site of fertilization [53]. Whether this predominant hypermethylation is directly related to or part of several complex mechanisms toward the success of fertilization remains to be studied. Of particular interest for pigs, being a polytocous species, was the hypomethylation found in *CLSTN2*, which is a candidate gene for litter size, as determined in sheep using GWAS [71]. 

The enrichment GO analysis showed an overrepresentation of SNPs in the pathways for the *GABRR2*, *PSPH*, *SLC35F3*, *PLA2G4A*, and *KATNAL1* genes. The first gene, the *GABRR2*, has been associated to social genetic effects for conversion rate in Yorkshire [72] and Landrace pigs [73]. The *KATNAL1* gene is essential for human spermiogenesis, meiosis, control of Sertoli cell microtubule dynamics [74], and being affected in cases of azoospermia [75] evidently related to male fertility. It is an inhibitor of human sperm maturation [76] and also a marker of puberty in Bama Xiang pigs [77]. Moreover, the modulation on its expression has been linked to sperm deformity in Chinese Holstein bulls [78]. In large white boars, the cytosolic phospholipase A2 group IV family A (*PLA2G4A*) is involved in cleaving arachidonic acid from phospholipids, being associated to motility, total sperm count, and morphological defects [18]. Overall, in any case, the changes in expression of genes related to motility adquisition and other associated events, as linked to the increased methylation in LF boars, should definitely be checked in future studies of sperm quality, attempting the establishment of still elusive biomarkers for male fertility.

## 4. Materials and Methods

### 4.1. Animals and Semen Collection, Evaluation, and Handling

Animal husbandry and all experimental and analytical procedures were performed in compliance with European Community and Swedish legislation (Directive 2010/63/EU; Swedish SJVFS 2017:40); and approvals were granted by the “Regional Committee for Ethical Approval of Animal Experiments” (Linköpings Djurförsöksetiska nämnd), Linköping, Sweden (Dnr 75-12; ID1400; 03416-2020); and the Bioethics Committee of Murcia University (research code: 639/2012).

Semen was collected from healthy and mature boars (1 to 2 years old) of Landrace and Large White breeds housed in an AI center belonging to the company Topigs Norsvin España (Spain) housed in climate-controlled barns, with free access to water and fed commercial feedstuff for adult boars. Boars were handled carefully and avoiding any unnecessary stress throughout and during manual (gloved hand method) collection of the sperm-rich fraction (SRF) of the ejaculate. The spermatozoa were separated from seminal plasma immediately after collection of the ejaculate by double centrifugation (1500× *g* at room temperature for 10 min using a Rotofix 32A, Hettich Zentrifugen, Tuttlingen, Germany) and the resulting sperm pellets were stored at −80 °C (Ultra Low Freezer; Haier Inc., Qingdao, China).

### 4.2. Experimental Design

A total of 39 ejaculates (SRF-spermatozoa) were collected from 11 AI boars over a period of four months, between August and November 2014 (late-summer: 1–15 August; early-autumn: 16 August–10 September; mid-autumn: 11 September–25 October and late-autumn: 26 October–19 December. The collected semen met quantity and quality pre-requirements for AI dose production e.g., ˃200 × 10^6^ spermatozoa/mL 70% motile spermatozoa and 75% morphologically normal spermatozoa. During the same period, semen AI doses (80 mL with 2.5 × 10^9^ spermatozoa) were produced using other ejaculates from the same boars and used in routine AI programs. Raw fertility records were obtained for 7 of the 11 boars, the remaining 4 boars thus marked as of Unknown Fertility (UF). These UF boars constituted a test group to explore whether they would be epigenetically similar to one of the groups with known High (HF) or Low (LF) Fertility. The raw fertility records were statistically corrected for farm and sow-related parameters in order to isolate the contribution of individual boars (direct boar effect) on fertility. This direct effect of the boar on fertility is the deviation of both the FR and the LS based on > 100 inseminated sows per boar with regard to the mean of the boar population of the same genetic line. The assessment led us to classify boars (*n* = 7) as having high (*n* = 4) or low (*n* = 3) fertility. High fertility boars showed a FR > 0.45 and a LS > 0.15, and those with low fertility FR < −0.12 and LS < −0.18.

### 4.3. Processing of the Spermatozoa for DNA Isolation

Semen samples were centrifuged twice (1500× g for 10 min at room temperature, Rotofix 32A, Hettich Zentrifugen, Tuttlingen, Germany), and the resulting sperm pellets were directly frozen in liquid nitrogen. The frozen-stored sperm pellets were re-suspended in 100 μL PBS and 100 μl of collagenase (850 u/mL) and incubated at 37 °C for 1 h under rotation. After incubation, the samples had 1 mL of PBS added and then were sonicated for 5 s at 60% amplitude (Fisher ultra-sonicator attached to a cooling chamber, cup horn, with capacity for eight microfuge tubes). The samples were subjected to three series of vertexing (30 s), centrifugation (4000 g, RT, 3 min), discarding of the supernatant, and re-suspension in 1 mL PBS (phosphate-buffered saline). However, the last re-suspension was done in 820 μL of digestion buffer (prepared by mixing 5 mL of 1 M Tris–HCl pH 8.0, 2 mL of 0.5 EDTA (ethylenediaminetetraacetic acid), 5 mL of 10% SDS (sodium dodecylsulfate) and 88 mL of DNAse free water). DTT (di-thio-treitol) was then added (80 μL; 0.1 M), and the mixture was incubated at 65 °C for 15 min.

Proteinase K (Sigma-Aldrich, St. Louis, MO, USA) was added to each sample (80 mL; 20 mg/mL), and incubation was performed under rotation for 1 h at 55 °C. After incubation, 300 μL of protein precipitation solution (Promega, Nacka, Sweden), was added and samples were incubated for 15 min on ice. Samples were centrifuged at max speed for 30 min at 4 °C, and then, 1 mL of the supernatant was transferred to a new tube. Then, isopropanol (1 mL, Sigma-Aldrich, St. Louis, MO, USA) and glycogen (3 μL; 5mg/mL, Sigma-Aldrich, St. Louis, MO, USA) were added. The samples were incubated at 4 °C under rotation for 30 min and then centrifuged at max speed for 30 min at 4 °C. Then, the supernatant was discarded, and 500 μL of 70% ethanol was added. Centrifugation was performed again for 10 min at 4 °C (ScanSpeed 1248B Labogene, Lillerød, Denmark). The supernatant was discarded and the samples were dried at the bench for at least 20 min; then, they were re-suspended with 150 μL of DNAse-free water. The DNA samples were quantified in a fluorometer (Qubit^®^ Fluorometric Quantitation). The DNA quality was evaluated using the Nanodrop^®^ 2000c spectrophotometer (Thermo Fisher Scientific, Waltham, MA, USA), and the integrity was checked on 1% agarose gels.

### 4.4. Preparation of Sequencing Libraries

The genome was first digested with the PstI restriction enzyme (Thermo Scientific; Waltham, MA, USA) in a suitable range (≈450 bp) for Illumina (San Diego, CA, USA) sequencing [26]. Then, illumina sequencing barcodes are ligated to each end of the digested DNA fragments, allowing the pool of DNA samples to be immunoprecipitated together. Each pooled DNA sample contains different barcodes identifying each individual reduced genome. Then, a 50 ng fraction of the DNA pool, representing the genetic background of the libraries, hereby called inputs, was amplified by PCR. Then, the methylated fraction of the sampled DNA was captured by an anti-methyl-cytosine antibody (Diagenode, Sparta, TN, USA) [29]. After this step, the methylated DNA was amplified using PCR, which is followed by a clean-up of the primer dimers and unbound adapters [79,80]. The procedure generated a library that corresponds to the methylated portion of the reduced genome. The libraries were quantified, clustered, and end-paired sequenced in the Illumina NovaSeq6000 platform with a read length of 150bp at the SNP & SEQ facilities of the SciLifeLab (Uppsala, Sweden).

### 4.5. Bioinformatics

Data were processed by CASAVA (Illumina) by converting “.bcl” (base calls) to “.fastq” extensions, compatible to other programs for reads alignment. Quality of short reads was checked with FastQC v.0.11.33. According to the pipeline used, quality trimming was performed using default parameters. Quality-trimmed reads for SNPs and DMRs were aligned to the current pig reference genome (Sus scrofa 11.1, NCSC) using default parameters for Bowtie2 tool v.2-2.3.4.3 [81]. The coverage depth of each sequenced file was determined using Samtools v.0.1.19 with the “depth” option. 

From the sequences generated by input sequencing, SNP call was executed by Tassel 5 v.2.0 [82], using the default TASSEL-GBS Discovery Pipeline. Criteria for inclusion were at least 1% for minimum minor allele frequency (mnMAF), 20% of minimum taxon (sample) coverage (mnTCov) and 90% for minimum site coverage (mnScov). SNPs that passed the filtering criteria were selected for GWAS using a weighted Mixed Linear Model (MLM) approach in which HF was set as case (affected) and LF was set as control (unaffected). For MLM, we used an optimum level of compression, and the variance component were re-estimated after each marker. We included a centered IBS kinship matrix in the model to be used as random effect (max alleles = 6). The model formula used can be accessed through the link: https://bitbucket.org/tasseladmin/tassel-5-source/wiki/UserManual/MLM/MLM (accessed on 20 January 2021). The Plink software v2 [83] was used to calculate the frequency of each allele, in the analyzed population, per treatment. 

For the methylation profile of the samples, Stacks v.2.41 was firstly used for data de-multiplexing [84] and the maintenance of quality-trimmed reads for sequences generated by the input and the methylation libraries sequenced, respectively. After that, as previously recommended [28], a pre-selection of the regions later used as input in MeDIPs was obtained by comparing the methylation enriched sets (Msets HF and LF) using the model-based analysis of ChIP-Seq data (MACS2) peak calling program (https://github.com/taoliu/MACS/, accessed on 2 February 2021) [85] with default parameters as described by [28]. Following read alignment and the peak calling, all analyses were performed using bioinformatics packages from the ‘R’ Bioconductor repository v. 3.6.1 [86]. The BSgenome.Sscrofa.UCSC.susScr11 package was uploaded as the reference genome. In MeDIPs, differences between the methylation enriched sets (Msets) were tested only on these MACS2-generated regions. MACS2 is a recommended tool to identify sample-wise “peak-specific” methylated regions of variable sizes in experiments using paired controls to determine enrichment against background [87,88,89]. The MeDIPs R-package was used for basic data processing, quality controls, normalization, and identification of differential coverage among the samples. The quality control was carried out to confirm the enrichment of the methylated fraction of the genome by calculating the average enrichment score (>1, around 2 being optimal) to denote methylated DNA enrichment [90]. In order to avoid possible artefacts caused by PCR amplification, MeDIPs allows a maximum number of stacked reads per genomic position. This is done by using a Poisson distribution of stacked reads genome-wide. A default threshold for the detection of stacked reads of *p* < 0.001 was used. Then, the reads that passed this quality control were standardized to 100 bp by extending smaller reads to this length, which is the paired-end read size generated by the Illumina NovaSeq platform. MeDIP-seq data were transformed into genome-wide relative methylation scores by a CpG-dependent normalization method [91]. This normalization is based on the dependency between short-read coverage and CpG density at genome-wide windows [92] and can be visualized as a calibration plot. A calibration plot was generated using one of the individuals that passed the cut-off index to generate a coupling set (object that groups information about CpG density genome-wide). Based on this, a threshold for a minimum sum of counts across all samples per window was defined (minRowSum = 1) to be further used as input for DMR though Limma package [93]. Then, sequencing data for each individual were assigned to one of the experimental groups, and differential coverage (i.e., DMR) was calculated. 

From these DMRs, the matrix containing only the list of read counts for each one of the regions of interest (ROIs) for each one of the individuals obtained by MeDIPs was extracted, followed by Linear Model testing, e.g., limma package from R for differential analysis of each one of the settled contrasts. 

The grouping of boars and lines based on the significant SNPs and DMRs found was obtained by PCA clustering and plotted in R using the plot function. The individuals of UF were only included in the PCA graph results. Then, these markers (SNPs and DMRs) were annotated against the pig reference genome (BSgenome.Sscrofa.UCSC.susScr11) using the annotatePeak function from the ChIPseeker package [94] in R environment. In this function, the txdb (as the transcript metadata) from GenomicFeatures package and org.Ss.eg.db package were used as the annotation database for the current pig genome. For the identification of affected molecular function, cellular components, and biological processes, the SNP and DMR associated genes were searched for Gene Ontology (http://geneontology.org, accessed on 14 February 2021) analysis through the enrichGO function within the ChIPseeker package [94]. For the identification of enriched molecular interaction and reaction networks, we used the Kyoto Encyclopedia of Genes and Genomes (KEGG; https://www.genome.jp/kegg, accessed on 15 February 2021), which was run with the enrichKegg function also within the ChIPseeker package [94].

An overlap analysis to identify DMRs overlapping with SNPs was performed by permutation tests (*n* = 100) to determine which peak overlaps were significant, using the findOverlapsOfPeaks function from the ChIPpeakAnno v3.6.5 R package with default parameters. Venn diagrams were plotted using the makeVennDiagram function within the same package.

### 4.6. Sequencing and Alignment

The average sequencing and alignment statistics for the individuals investigated in the experiment are shown in Table 5. More details can be found in Supplementary Table S2. We sequenced both the reduced genome and its methylated fraction. For genetic and epigenetic differential studies, we did not use the UF boars, they were used only in the posterior analysis. The reduced genome obtained through the GBS approach was used as the input for genetic background analyses. For that, we used the merged .bam file from the biological replicates (*n* = 11, being 4HF, 3LF, and 4UF), while for the methylation analysis, we considered replicates in the model (*n* = 39, being 15HF, 11LF, and 13UF), since multiple ejaculates were collected in different periods.

## 5. Conclusions

This paper describes potential candidate genes for fertility diagnosis across populations of breeding boars. Particular genes, *ROPN1L*, *KIF2B*, *LMX1A*, and *FOX11*, related to 46 DMRs, were observed in the high- and low-fertility populations analyzed here. Among the gene-related DMRs obtained, we also found the genes *GABRR2*, *PSPH*, *SLC35F3*, *PLA2G4A*, and *KATNAL1*, which are related to the top two biological pathways enriched in SNPs both high- and low-fertility groups. In addition, we provide a robust list of DMRs related to a condition dependent on a wide range of determinants for fertility. The biological functions of the genes associated to the DMRs found in our study, and their related enriched pathways, are relevant for sperm function. DMRs could be tested more extensively as markers for fertility diagnosis in order to monitor different animal production setups. In conclusion, fertility levels in breeding males including farrowing rate (FR) and litter size (LS) can be discerned using methylome analyses, and the findings in this biomedical animal model ought to be applied, besides for sire selection, for andrological diagnosis of idiopathic sub/infertility. 

## Figures and Tables

**Table 1:** Five distinct contrasts used through a linear model to compare the phenotypic breeding boar groups.

Contrast	Group1	Group2	Purpose of Contrast
**HvL**	HF	LF	to compare genomic data between HF and LF individuals
**H_LSvsMA**	H_LS	H_MA	to compare the effect of the sampling date between late-summer (LS) and mid-autumn (MA) on HF individuals
**L_LSvsMA**	L_LS	L_MA	to compare the effect of the sampling date between late-summer (LS) and mid-autumn (MA) on LF individuals
**HvsL_LS**	H_LS	L_LS	to compare genomic data between HF and LF individuals at the LS sampling period
**HvsL_MA**	H_MA	L_MA	to compare genomic data between HF and LF individuals at the MA sampling period

HF = high fertility, LF = low fertility, LS = late-summer, LA = late-autumn, and MA = mid-autumn.

**Table 2:** Genomic features and annotation of the TOP-SNPs identified between HF and LF boars using Genomic-Wide Association Studies (GWAS) (the CG seqCpG is marked in red).

Location	Site Summary in the Analyzed Population *n* = 7						Annotation					
	Ref	Alt	A1	F_A1 HF	F_A 1 LF	A2	SeqCpG	Annotation	Distance to TSS	ENSEMBL ID	*GENE ID*	Description
chr1:13745053	G	A	A	0.5	0.25	G	GC	Intron	28,711	ENSSSCG00000004081		
chr1:13745059	T	C	C	0.5	0.25	T	TC		28,717			
chr1:13745092	C	T	T	0.5	0.25	C	CG		28,750			
chr1:54055492	A	G	G	0	0.625	A	AG	Distal Intergenic	33,321	ENSSSCG00000035731	*−*	
chr1:54055493	G	C	C	0	0.625	G	GC		33,320			
chr1:54055625	G	C	C	0	0.625	G	GA		33,188			
chr1:57269839	T	A	A	0	0.625	T	TG	Intron	−3712	ENSSSCG00000024249	*GABRR2*	gamma-aminobutyric acid type A receptor subunit rho2 [Source:NCBI gene (formerly Entrezgene);Acc:100522289]
chr1:57269858	T	C	C	0	0.625	T	TC		−3731			
chr1:57277143	C	A	A	0	0.625	C	CA		−11,016			
chr1:57528770	G	A	A	0	0.625	G	GC	Intron	61,949	ENSSSCG00000050825		
chr1:57621352	G	A	A	0	0.625	G	GC	Promoter	2902	ENSSSCG00000004322	*ANKRD6*	ankyrin repeat domain 6 [Source:NCBI gene (formerly Entrezgene);Acc:102167335]
chr1:57621367	G	A	A	0	0.625	G	GG		2917			
chr1:57621370	T	C	C	0	0.625	T	TT		2920			
chr1:128707723	C	T	T	0	0.625	C	TG	Promoter	1229	ENSSSCG00000022039		
chr1:140846691	A	C	C	0.5	0.25	A	AT	Intron	170,343	ENSSSCG00000038547	*GABRB3*	gamma-aminobutyric acid type A receptor subunit beta3 [Source:VGNC Symbol;Acc:VGNC:88308]
chr1:167452422	C	G	G	0	0.625	C	GA	Distal Intergenic	63,364	ENSSSCG00000045133		
chr1:168947778	T	G	G	0	0.625	T	TG	Intron	−147,253	ENSSSCG00000045715		
chr2:10168387	C	T	C	0	0.625	T	TC	Intron	3002	ENSSSCG00000013087	*TKFC*	triokinase and FMN cyclase [Source:NCBI gene (formerly Entrezgene);Acc:100520121]
chr2:10168389	C	G	G	0	0.625	C	CA		3000			
chr2:13387750	G	T	T	0	0.625	G	TG	Promoter	−2942	ENSSSCG00000031163		smoothelin like 1 [Source:NCBI gene (formerly Entrezgene);Acc:110259259]
chr2:13387762	G	A	A	0	0.625	G	AA		−2954			
chr3:16873170	G	C	C	0.5	0.25	G	GT	Distal Intergenic	−4753	ENSSSCG00000007748	*PSPH*	phosphoserine phosphatase [Source:VGNC Symbol;Acc:VGNC:91931]
chr6:3925771	C	G	G	0	0.625	C	CA	Intron	18,808	ENSSSCG00000002669	*CRISPLD2*	cysteine rich secretory protein LCCL domain containing 2 [Source:VGNC Symbol;Acc:VGNC:96958]
chr6:3925776	C	G	G	0	0.625	C	CC		18,803			
chr6:3925779	C	T	T	0	0.625	C	CC		18,800			
chr6:7702343	G	C	C	0	0.625	G	GG	Intron	−4397	ENSSSCG00000045637		
chr6:7702375	G	A	A	0	0.625	G	GG		−4429			
chr6:101129470	A	G	G	0	0.625	A	AT	Distal Intergenic	35,288	ENSSSCG00000049298		
chr7:87106399	G	T	T	0.5	0.25	G	GA	Distal Intergenic	−3090	ENSSSCG00000002263	*SLCO3A1*	solute carrier organic anion transporter family member 3A1 [Source:VGNC Symbol;Acc:VGNC:93197]
chr7:115966284	C	T	T	0	0.625	C	CG	Intron	5808	ENSSSCG00000030371	*SERPINA3-2*	alpha-1-antichymotrypsin 2 [Source:NCBI gene (formerly Entrezgene);Acc:396686]
chr7:115966307	C	T	T	0	0.625	C	CC		5785			
chr7:115982628	C	A	A	0	0.625	C	AA		4776			
chr7:115982655	G	T	T	0	0.625	G	TG		4749			
chr8:1318578	C	T	T	0.5	0.25	C	CG	Intron	5424	ENSSSCG00000034633	*CFAP99*	cilia and flagella associated protein 99 [Source:VGNC Symbol;Acc:VGNC:86608]
chr8:1328667	A	G	G	0.5	0.25	A	AG		15,513			
chr9:40003949	A	G	G	0.5	0.25	A	GC	Distal Intergenic	−46,046	ENSSSCG00000050685		
chr9:40003978	T	C	C	0.5	0.25	T	TG		−46,017			
chr9:45947327	G	T	T	0.5	0.25	G	TT	Intron	15,089	ENSSSCG00000028204	*TREH*	trehalase [Source:VGNC Symbol;Acc:VGNC:94382]
chr11:7034734	A	T	T	0.5	0.25	A	AA	Intron	50,584	ENSSSCG00000009326	*KATNAL1*	katanin catalytic subunit A1 like 1 [Source:VGNC Symbol;Acc:VGNC:89310]
chr11:16127229	G	A	A	0.5	0.25	G	GG	Intron	67,905	ENSSSCG00000040538	*WDFY2*	WD repeat and FYVE domain containing 2 [Source:VGNC Symbol;Acc:VGNC:94902]
chr11:61861680	G	C	C	0	0.625	G	GG	Intron	323,962	ENSSSCG00000048281		
chr12:19677123	G	A	A	0	0.625	G	GC	Intron	−4345	ENSSSCG00000040316	*U2*	U2 spliceosomal RNA [Source:RFAM;Acc:RF00004]
chr12:41648810	G	A	A	0.5	0.25	G	GT	Intron	−78,192	ENSSSCG00000040177	*ASIC2*	acid sensing ion channel subunit 2 [Source:VGNC Symbol;Acc:VGNC:97893]
chr12:41648838	T	C	C	0.5	0.25	T	TC		−78,164			
chr12:42246850	A	G	G	0	0.625	A	AA	Intron	−61,012	ENSSSCG00000050001		
chr14:4991961	T	G	G	1	0.125	T	TT	Distal Intergenic	419,328	ENSSSCG00000018795		
chr14:24479770	A	G	G	0	0.625	A	GT	Intron	46,536	ENSSSCG00000009748	*RIMBP2*	RIMS binding protein 2 [Source:VGNC Symbol;Acc:VGNC:92306]
chr14:56252347	G	C	C	0.5	0.25	G	GG	Promoter	−1423	ENSSSCG00000043646		
chr15:63579255	C	T	T	0.5	0.25	C	CC	Distal Intergenic	−13,443	ENSSSCG00000015872	*GPD2*	glycerol-3-phosphate dehydrogenase 2 [Source:VGNC Symbol;Acc:VGNC:96330]
chr15:63579295	A	G	G	0.5	0.25	A	AA		−13,403			
chr15:140048049	C	T	T	0.5	0.25	C	CG	Intron	−12,312	ENSSSCG00000016368	*FARP2*	FERM, ARH/RhoGEF and pleckstrin domain protein 2 [Source:VGNC Symbol;Acc:VGNC:95809]
chr15:140089430	A	T	T	0.5	0.25	A	AA		14,636			
chr16:53406651	T	C	C	0	0.625	T	CC	Intron	−81,737	ENSSSCG00000017003	*KCNIP1*	potassium voltage-gated channel interacting protein 1 [Source:VGNC Symbol;Acc:VGNC:89348]
chrY:5970972	T	A	T	1	0	A	AA	Intron	24,002	ENSSSCG00000039894		
chrY:5970979	G	C	G	1	0	C	CG		24,009			

**Table 3:** Tested contrasts and numbers of DMRs obtained using two different thresholds.

Contrasts	Number of DMRs (*p* ≤ 0.05)	Number of DMRs (FDR ≤ 0.5)
HF–LF	46	0
LS–LA	40	0
MA–LA	41	0
MA–LS	49	0
HF_LS–HF_MA	87	7
LF_LS–LF_MA	27	0
HF_LS–LF_LS	48	4
HF_MA–LF_MA	62	3

Contrasts: HF = high fertility, LF = low fertility, LS = late-summer, LA = late-autumn, and MA = mid-autumn.

**Table 4:** Descriptive statistics and gene annotations for the significant fertility-related DMRs.

Location	logFC	*p*-Value	adj.*p*.Val	Annotation	Distance ToTSS	EMSEMBL ID	*GENE ID*	Description	CG
chr1:35377643-35377942	1.20	1.86 × 10^−2^	9.96 × 10^−1^	Intron	44,178	ENSSSCG00000024392	*THEMIS*	thymocyte selection associated (Source:VGNC Symbol;Acc:VGNC:98369)	9
chr1:71457907-71458215	1.10	4.88 × 10^−2^	9.96 × 10^−1^	Promoter	−502	ENSSSCG00000045756			8
chr1:89431929-89432118	1.63	1.54 × 10^−2^	9.96 × 10^−1^	Distal Intergenic	−167,999	ENSSSCG00000046698			1
chr1:122165935-122166122	0.93	1.34 × 10^−2^	9.96 × 10^−1^	Intron	26,493	ENSSSCG00000004648	*FAM227B*	family with sequence similarity 227 member B (Source:VGNC Symbol;Acc:VGNC:87961)	8
chr2:15503913-15504126	1.42	6.22 × 10^−3^	9.40 × 10^−1^	Intron	27,523	ENSSSCG00000020542	*U6*	U6 spliceosomal RNA (Source:RFAM;Acc:RF00026)	10
chr2:65812355-65812642	1.18	2.39 × 10^−2^	9.96 × 10^−1^	Intron	90,547	ENSSSCG00000013754		calcium voltage-gated channel subunit alpha1 A (Source:VGNC Symbol;Acc:VGNC:99705)	6
chr2:72272984-72273185	1.35	4.28 × 10^−2^	9.96 × 10^−1^	5’ UTR	−3761	ENSSSCG00000013556		adhesion G protein-coupled receptor E1 (Source:VGNC Symbol;Acc:VGNC:85124)	21
chr3:38143485-38143693	1.11	1.99 × 10^−2^	9.96 × 10^−1^	Intron	−29,370	ENSSSCG00000007950	*ADCY9*	adenylate cyclase 9 (Source:VGNC Symbol;Acc:VGNC:85113)	6
chr3:111685876-111686073	1.76	1.24 × 10^−2^	9.96 × 10^−1^	Promoter	2167	ENSSSCG00000026367	*IFT172*	intraflagellar transport 172 (Source:VGNC Symbol;Acc:VGNC:89046)	2
chr3:128777380-128777600	−1.13	4.46 × 10^−2^	9.96 × 10^−1^	Intron	8370	ENSSSCG00000008646	*RNF144A*	ring finger protein 144A (Source:VGNC Symbol;Acc:VGNC:92358)	2
chr4:68193523-68193723	−1.13	2.97 × 10^−2^	9.96 × 10^−1^	Distal Intergenic	31,282	ENSSSCG00000023848	*VXN*	vexin (Source:VGNC Symbol;Acc:VGNC:94889)	4
chr4:85493823-85493995	−0.98	4.74 × 10^−2^	9.96 × 10^−1^	Intron	86,294	ENSSSCG00000006329	*LMX1A*	LIM homeobox transcription factor 1 alpha (Source:VGNC Symbol;Acc:VGNC:89770)	6
chr6:19702050-19702740	1.18	4.64 × 10^−2^	9.96 × 10^−1^	Intron	−5977	ENSSSCG00000002812	*KIFC3*	kinesin family member C3 (Source:VGNC Symbol;Acc:VGNC:89479)	8
chr6:112264267-112264451	0.78	2.87 × 10^−2^	9.96 × 10^−1^	Distal Intergenic	259,145	ENSSSCG00000042906			8
chr7:99786455-99786655	1.23	4.55 × 10^−2^	9.96 × 10^−1^	Distal Intergenic	−57,879	ENSSSCG00000051045			1
chr7:104533019-104533586	1.10	2.47 × 10^−2^	9.96 × 10^−1^	Promoter	−284	ENSSSCG00000049060			16
chr9:19560588-19560803	1.39	1.62 × 10^−2^	9.96 × 10^−1^	Distal Intergenic	12,942	ENSSSCG00000014905	*TMEM126B*	transmembrane protein 126B (Source:NCBI gene (formerly Entrezgene);Acc:100626990)	6
chr9:91128754-91129011	1.85	1.65 × 10^−2^	9.96 × 10^−1^	Intron	70,639	ENSSSCG00000015383	*RAPGEF5*	Rap guanine nucleotide exchange factor 5 (Source:VGNC Symbol;Acc:VGNC:92094)	1
chr10:19289977-19290168	1.28	2.26 × 10^−2^	9.96 × 10^−1^	Distal Intergenic	−16,228	ENSSSCG00000033907			5
chr11:1065989-1066196	1.29	2.33 × 10^−2^	9.96 × 10^−1^	3’ UTR	28,854	ENSSSCG00000009276	*XPO4*	exportin 4 (Source:VGNC Symbol;Acc:VGNC:95004)	1
chr11:1065989-1066196	1.29	2.33 × 10^−2^	9.96 × 10^−1^	3’ UTR	28,854				1
chr11:19350117-19350833	1.00	2.26 × 10^−2^	9.96 × 10^−1^	Intron	5261	ENSSSCG00000009403	*ITM2B*	integral membrane protein 2B (Source:NCBI gene (formerly Entrezgene);Acc:595120)	12
chr12:30052250-30052448	1.02	4.87 × 10^−2^	9.96 × 10^−1^	Distal Intergenic	−109,077	ENSSSCG00000017599	*KIF2B*	kinesin family member 2B (Source:VGNC Symbol;Acc:VGNC:89468)	3
chr12:53660655-53660898	−1.18	3.90 × 10^−2^	9.96 × 10^−1^	Exon	−50,792	ENSSSCG00000039032	*U6*	U6 spliceosomal RNA (Source:RFAM;Acc:RF00026)	16
chr12:59826222-59826441	1.56	2.74 × 10^−3^	6.80 × 10^−1^	Intron	19,267	ENSSSCG00000018045	*ULK2*	unc-51 like autophagy activating kinase 2 (Source:VGNC Symbol;Acc:VGNC:94695)	6
chr13:7508-7838	1.18	3.37 × 10^−3^	6.80 × 10^−1^	Distal Intergenic	281,632	ENSSSCG00000046931			9
chr13:34107-34423	0.89	2.52 × 10^−2^	9.96 × 10^−1^	Distal Intergenic	255,047				5
chr13:37450-37723	1.03	2.98 × 10^−2^	9.96 × 10^−1^	Distal Intergenic	251,747				5
chr13:42463-42749	1.40	4.72 × 10^−3^	8.14 × 10^−1^	Distal Intergenic	246,721				7
chr13:83112-83393	1.21	1.35 × 10^−2^	9.96 × 10^−1^	Distal Intergenic	206077				5
chr13:96003-97046	1.06	3.01 × 10^−3^	6.80 × 10^−1^	Distal Intergenic	192,424				18
chr13:104471-105251	0.89	2.12 × 10^−2^	9.96 × 10^−1^	Distal Intergenic	184,219				12
chr13:109921-111269	0.79	3.21 × 10^−2^	9.96 × 10^−1^	Distal Intergenic	178,201				27
chr13:120981-122627	0.92	2.74 × 10^−2^	9.96 × 10^−1^	Distal Intergenic	166,843				34
chr13:133660-134567	1.30	2.51 × 10^−3^	6.80 × 10^−1^	Distal Intergenic	154,903				22
chr13:138579-139179	1.02	2.88 × 10^−2^	9.96 × 10^−1^	Distal Intergenic	150,291				10
chr13:80744108-80744758	−1.08	3.46 × 10^−2^	9.96 × 10^−1^	Distal Intergenic	−44,636	ENSSSCG00000011666	*CLSTN2*	calsyntenin 2 (Source:VGNC Symbol;Acc:VGNC:86785)	6
chr15:34797-35849	−1.02	3.24 × 10^−2^	9.96 × 10^−1^	Distal Intergenic	−4170	ENSSSCG00000047217			23
chr15:134010175-134010376	1.18	4.26 × 10^−2^	9.96 × 10^−1^	Intron	−4041	ENSSSCG00000049095			5
chr16:8511-12498	1.10	1.66 × 10^−3^	6.80 × 10^−1^	Distal Intergenic	−30,855	ENSSSCG00000022306	*ROPN1L*	rhophilin associated tail protein 1 like (Source:VGNC Symbol;Acc:VGNC:92406)	98
chr16:13425-15353	0.77	1.10 × 10^−2^	9.96 × 10^−1^	Distal Intergenic	−28,000				45
chr16:16135-17774	0.70	2.72 × 10^−2^	9.96 × 10^−1^	Distal Intergenic	−25,579				41
chr16:19241-21132	0.80	8.75 × 10^−3^	9.96 × 10^−1^	Distal Intergenic	−22,221				44
chr16:21825-23929	0.77	1.18 × 10^−2^	9.96 × 10^−1^	Distal Intergenic	−19,424	ENSSSCG00000022306	*ROPN1L*	rhophilin associated tail protein 1 like (Source:VGNC Symbol;Acc:VGNC:92406)	50
chr16:53839747-53839973	2.25	6.94 × 10^−4^	6.80 × 10^−1^	Promoter	−638	ENSSSCG00000017009	*FOXI1*	forkhead box I1 (Source:VGNC Symbol;Acc:VGNC:88208)	14
chr17:36220445-36220684	1.04	4.29 × 10^−2^	9.96 × 10^−1^	Intron	−19,300	ENSSSCG00000007249	*NOL4L*	COMM domain containing 7 (Source:NCBI gene (formerly Entrezgene);Acc:100514995)	7
chr17:36220445-36220684	1.04	4.29 × 10^−2^	9.96 × 10^−1^	Intron	−19,300				7
chr18:1373929-1374129	1.09	3.31 × 10^−2^	9.96 × 10^−1^	Intron	12,247	ENSSSCG00000037841	*ssc-mir-153*	ssc-mir-153 (Source:miRBase;Acc:MI0002454)	7

**Table 5:** Average sequencing and alignment statistics for individual boars.

Samples	Treat.	Sample Number	Depth ± SD			Number of bp Sequenced ± SD			Breadth (bp Sequenced/Depth) ± SD			% of the *% of Susscr11.1* Covered ± SD		
**GBS**	All	11	33.81	±	13.38	2746210261	±	1651232585	72531345	±	24303149	2.93	±	0.98
	Hi	4	34.92	±	15.21	2979989907	±	1926403257	75972199	±	28862451	3.07	±	1.16
	Low	3	35.98	±	11.79	2875589615	±	1539581798	75762826	±	19130954	3.06	±	0.77
	UF	4	31.09	±	16.00	2415396101	±	1887272897	66666880	±	28630213	2.69	±	1.15
**GBS + MEDIP**	All	39	19.30	±	2.35	128051103	±	35431948	6721573	±	1830176	0.27	±	0.07
	Hi	15	19.72	±	2.79	136199403	±	49129755	7021683	±	2391520	0.28	±	0.10
	Low	11	19.07	±	1.38	117088079	±	24901730	6219760	±	1640964	0.25	±	0.07
	UF	13	19.01	±	2.55	127925623	±	21202711	6799904	±	1157695	0.27	±	0.05

GBS = genotyping by sequencing, MEDIP = methylated DNA immunoprecipitation technique.

## Data Availability

The dataset supporting the conclusions of this article is available from the European Nucleotide Archive (ENA) repository (EMBL-EBI), under accession number PRJEB43108 (www.ebi.ac.uk/ena/data/view/PRJEB43108, accessed on 14 January 2021).

## Supplementary Materials

The following are available online at https://www.mdpi.com/1422-0067/22/5/2679/s1: Figure S1: Principal Component Analysis (PCA) of 42,963 SNPs (a) and those significant (*p* < 0.0005) SNPs (b) across 11 individual boars classified in experimental as High Fertile (HF), Low Fertile (LF), or with Unknown Fertility (UF), Figure S2: Principal Component Analysis (PCA) of the significant Differentially Methylated Regions (DMRs, voom-normalized methylation) of the ejaculates from all 11 boars grouped by (a): collection code, (b): collection period and, (c) collection week, Figure S3: Vulcano plots depicting differences in fertility-related DMRs found between ejaculates collected during late summer (LS) vs. middle autumn (MA) in (a) high-fertile (HF) boars, (b) low-fertile (LF); or (c) HF vs. LF boars collected during LS, or (d) during MA, Spreadsheet S1: Statistics and annotations performed for each one of the identified SNPs in this study, Spreadsheet S2: Phenotype information for each of the collected samples used in this study, Spreadsheet S3: Statistics and annotations performed for each one of the regions of interest analyzed in this study, Spreadsheet S4: GO enrichment output from the significant SNP- and DMR-related genes identified in this study, Table S1. Number of reads for each of the 11 individuals and their replicates, Table S2: Sequencing and alignment statistics for the individuals in the experiment.
