# Supplementary material for: Sperm Methylome Profiling Can Discern Fertility Levels in the Porcine Biomedical Model
Source: Int J Mol Sci. 2021 Mar 6;22(5):2679. doi: 10.3390/ijms22052679 (PMC7961483; doi:10.3390/ijms22052679)
Supplement: Supplementary file 1 [file ijms-22-02679-s001.zip › supp for conversion final/SupplementaryFiguresS1 S2 S3.pdf]

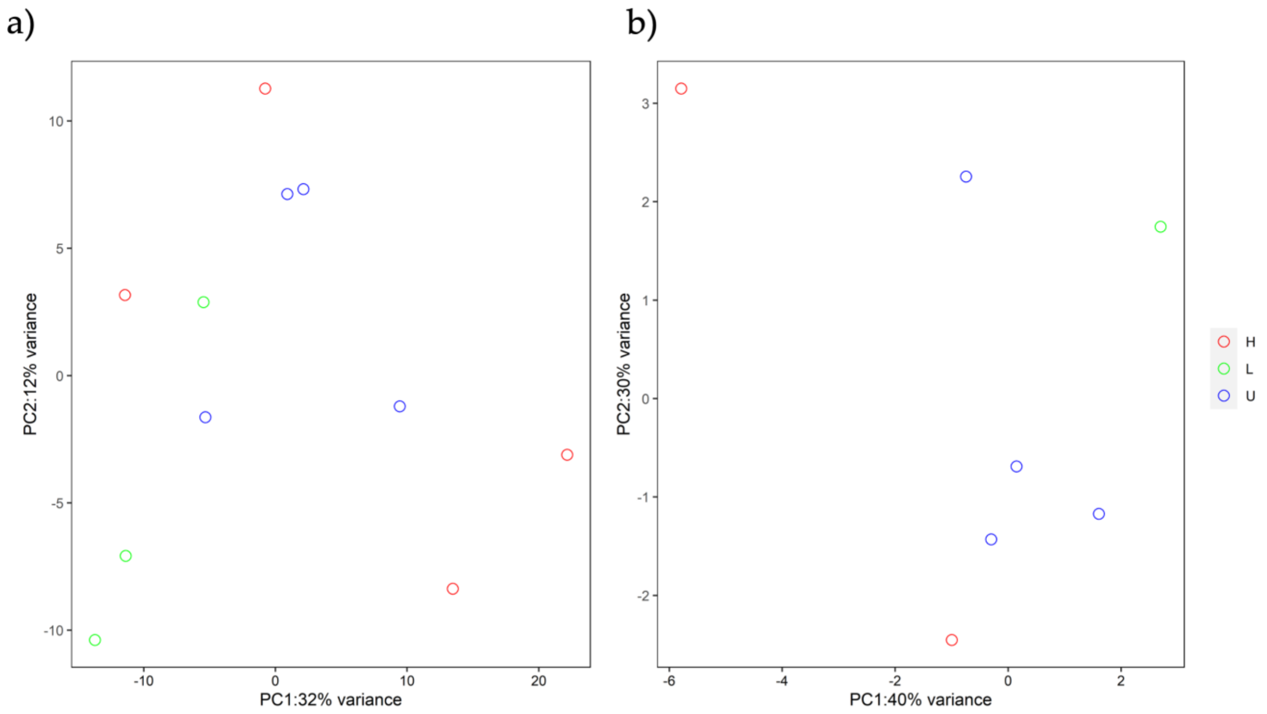

P≤ 0.0005 P≤0.000025

Figure S1. Principal Component Analysis (PCA) of 42,963 SNPs (**a**) and those significant ($p < 0.0005$) SNPs (**b**) across 11 individual boars classified in experimental as High Fertile (HF), Low Fertile (LF), or with Unknown Fertility (UF).

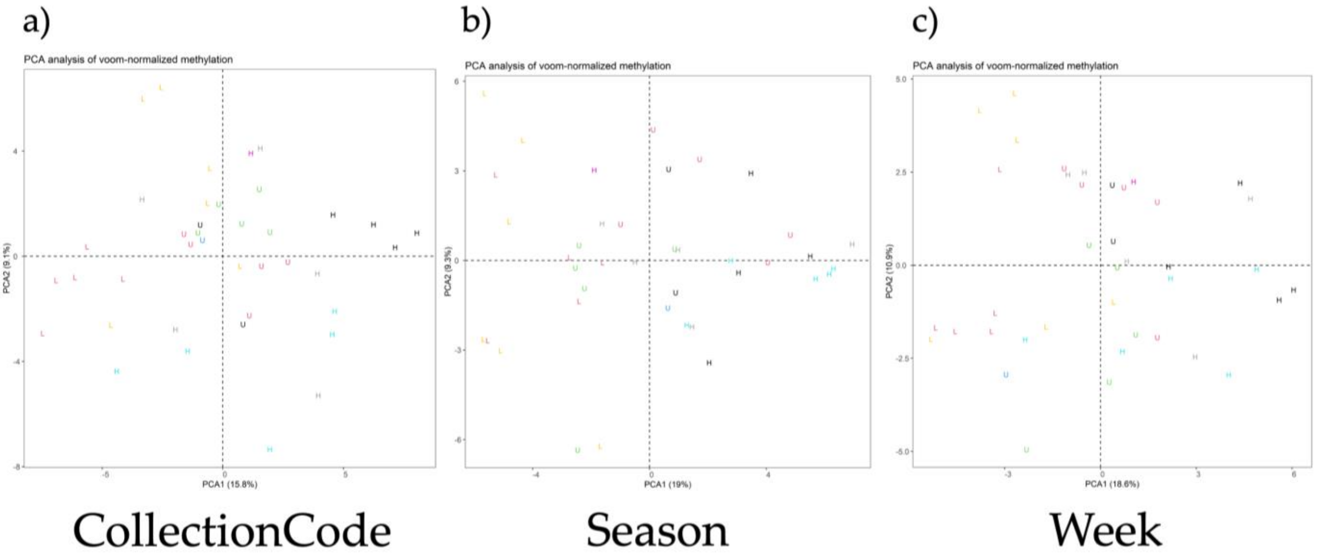

Figure S2. Principal Component Analysis (PCA) of the significant Differentially Methylated Regions (DMRs, voom-normalized methylation) of the ejaculates from all 11 boars grouped by (**a**): collection code, (**b**): collection period and, (**c**) collection week.

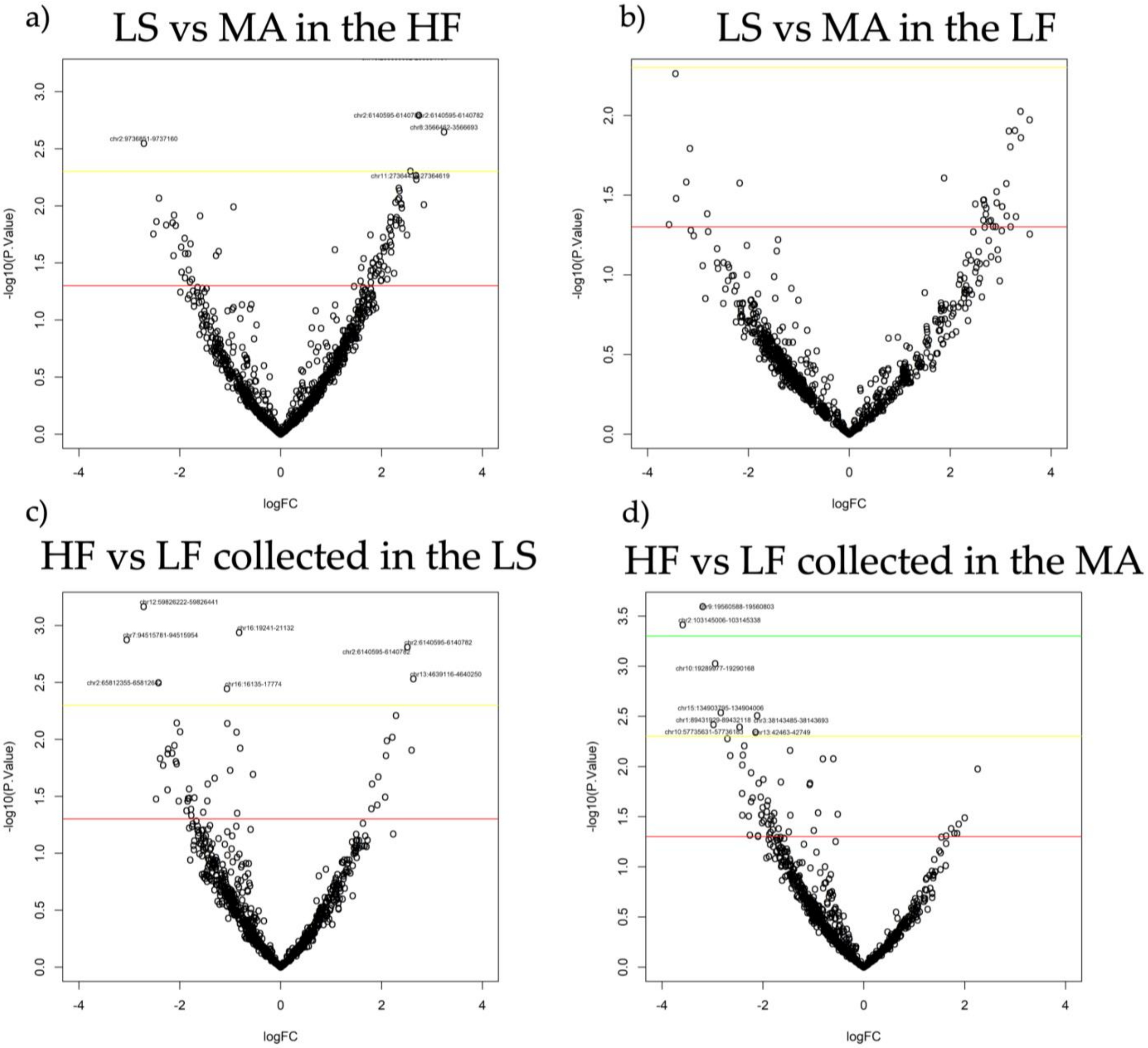

Figure S3. Vulcano plots depicting differences in fertility-related DMRs found between ejaculates collected during late summer (LS) vs. middle autumn (MA) in (**a**) high-fertile (HF) boars, (**b**) low-fertile (LF); or (**c**) HF vs. LF boars collected during LS, or (**d**) during MA.
